# Study on the Interaction of a Peptide Targeting Specific G-Quadruplex Structures Based on Chromatographic Retention Behavior

**DOI:** 10.3390/ijms24021438

**Published:** 2023-01-11

**Authors:** Ju Wang, Junqin Qiao, Weijuan Zheng, Hongzhen Lian

**Affiliations:** 1 State Key Laboratory of Analytical Chemistry for Life Science, School of Chemistry & Chemical Engineering and Center of Materials Analysis, Nanjing University, 163 Xianlin Avenue, Nanjing 210023, China; 2 State Key Laboratory of Pharmaceutical Biotechnology, School of Life Sciences, Nanjing University, 163 Xianlin Avenue, Nanjing 210023, China

**Keywords:** size exclusion chromatography, G-quadruplexes, G4-peptide interaction, retention behavior, conformational selectivity

## Abstract

G-quadruplexes (G4s) are of vital biological significance and G4-specific ligands with conformational selectivity show great application potential in disease treatment and biosensing. RHAU, a RNA helicase associated with AU-rich element, exerts biological functions through the mediation of G4s and has been identified to be a G4 binder. Here, we investigated the interactions between the RHAU peptide and G4s with different secondary structures using size exclusion chromatography (SEC) in association with circular dichroism (CD), ultraviolet-visible (UV-Vis) absorption, and native polyacrylamide gel electrophoresis (Native-PAGE). Spectral results demonstrated that the RHAU peptide did not break the main structure of G4s, making it more reliable for G4 structural analysis. The RHAU peptide was found to display a structural selectivity for a preferential binding to parallel G4s as reflected by the distinct chromatographic retention behaviors. In addition, the RHAU peptide exhibited different interactions with intermolecular parallel G4s and intramolecular parallel G4s, providing a novel recognition approach to G4 structures. The findings of this study enriched the insight into the binding of RHAU to G4s with various conformations. It is noteworthy that SEC technology can be easy and reliable for elucidating G4–peptide interactions, especially for a multiple G4 coexisting system, which supplied an alternative strategy to screen novel specific ligands for G4s.

## 1. Introduction

Besides the classic Watson–Crick double helix, nucleic acids strands can also fold into non-classical secondary structures, such as G-quadruplexes [1] and i-motifs [2]. Among these, G-quadruplexes (G4s), a four-stranded structure formed by guanine-rich (G-rich) sequences, have been extensively investigated due to their important biological relevance and application potential. G4 is composed of stacked G-tetrad layers, each of which is formed by four guanine bases in a coplanar arrangement through Hoogsteen hydrogen bonds. Cations stabilize G4 structures by coordinating electronegative carbonyl groups of guanines to reduce the repulsion between oxygen atoms of different G-tetrads [3]. Studies have found that G4s are highly polymorphic [4,5,6]. Based on strand arrangement orientation, G4 polymorphism can be manifested as different topologies, i.e., parallel, antiparallel and hybrid topology [7]. In addition, G4 polymorphism can also be reflected in the formation of G4 dimers, trimers and higher-ordered structures, which is also called G4 multimerization [8]. Moreover, based on the number of nucleic acid strands, G4s can be classified into unimolecular (intramolecular), bimolecular and tetramolecular (intermolecular) G4s, which is also considered to be a manifestation of G4 polymorphism.

So far, the existence of G4s in living cells has been demonstrated [9,10]. Further studies verified the essential role of G4s in gene expression [11,12] and in disease diagnosis and therapies [13]. Because of the ubiquity of G4s in the promoter regions of disease-related genes [14,15] and human telomeric regions [16], G4 ligands display great potential in disease treatment and biological process regulation. Many G4 ligands have been developed or discovered, which can be divided into chemical small molecule ligands [17,18,19,20,21,22,23] and biological macromolecule ligands [24,25,26,27]. RHAU (DHX36 or G4R1), a RNA helicase associated with AU-rich element, having significant biological functions in the regulation of telomerase [28], heart development [29] and gene expression [30], can specifically recognize G4s [31,32]. The conserved RHAU-specific motif (RSM) domain of RHAU is mainly responsible for the affinity to G4s [33]. RHAU peptide is a promising tool to target G4s, which possesses the recognizing ability without unwinding G4s. A minimal 18-amine-acid peptide containing an RSM domain is sufficient for recognizing G4s [26] and longer peptides display higher affinity due to additional interactions [34,35]. In current RHAU–G4-related studies, G4s involved are usually single in type. In addition, the research focus is mainly on the recognition mechanism, structural basis [36,37] and the unwinding activity of RHAU [34,38,39], where nuclear magnetic resonance and electrophoresis technologies are mostly applied. Are there other methods can be used for elucidating the G4–RHAU interaction? Additionally, relatively little is known about the interaction between RHAU and different G4 structures, especially when multiple G4 structures coexist. Therefore, how to easily study the interaction between RHAU and different G4 structures deserves in-depth investigation.

Chromatography is a traditional separation technology. The critical demand for high-resolution separation in biomedical research [40,41] has been driving the improvement of chromatography, especially liquid chromatography. Besides the conventional capabilities, chromatography can also be used for the characterization of advanced material properties [42], the elucidation of interaction mechanisms between materials and analytes [43] and the like, showing the unlimited potential of this 100-year-old technology. High-performance liquid chromatography (HPLC) is a powerful tool for the separation and analysis of complex systems. Compared with other methods, HPLC is easy to operate, stable, reliable, and can be used for simultaneous separation and analysis. In recent years, HPLC has gradually shown great potential in the analysis of G4s [44,45,46,47], making it a good alternative method for G4 studies. Among various chromatographic modes, size exclusion chromatography (SEC) is mostly used because the mobile phase used in SEC is most favorable to stabilize G4 structures. Miller et al. exploited SEC to analyze the polymorphism of oncogene promoter G4-forming sequences [48]. Marzano et al. used SEC to investigate the species of multimers [49] and G-wire nanostructures [50] of G4-forming oligodeoxynucleotides. Analysis via SEC facilitates research on G4 structures and biophysical properties without the need of sequence modifications, which is beneficial for investigating G4s formed under physiological conditions. Moreover, SEC can also be used to assess the interaction between ligands and G4s. Benito et al. utilized SEC to study the interaction between crystal violet and three G-rich sequences with the capacity to form multimers, and the change in peak intensity was used as the indicator to evaluate the interactions [51]. 

The retention behavior of solutes in chromatography is closely related to their structures. Since RHAU and G4 are both macromolecules, the retention of G4–RHAU complexes should be different from that of G4s in SEC. Therefore, the change in retention behaviors in SEC can be used for reflecting the G4–RHAU interactions. In this article, the interactions between RHAU peptide (hereafter referred to simply as RHAU) and hybrid, antiparallel, parallel and mixed G4s were investigated using SEC, combined with circular dichroism (CD) and ultraviolet-visible (UV-Vis) absorptions. Native polyacrylamide gel electrophoresis (Native-PAGE) was used for SEC result verification. Based on this study, the difference in binding ability of RHAU to various G4 structures was revealed clearly, which provided a simple and reliable approach for research into the interaction between peptide and G4s, especially for a multiple G4 coexisting system.

## 2. Results and Discussion

### 2.1. CD and UV-Vis Analysis

**Figure 1:**
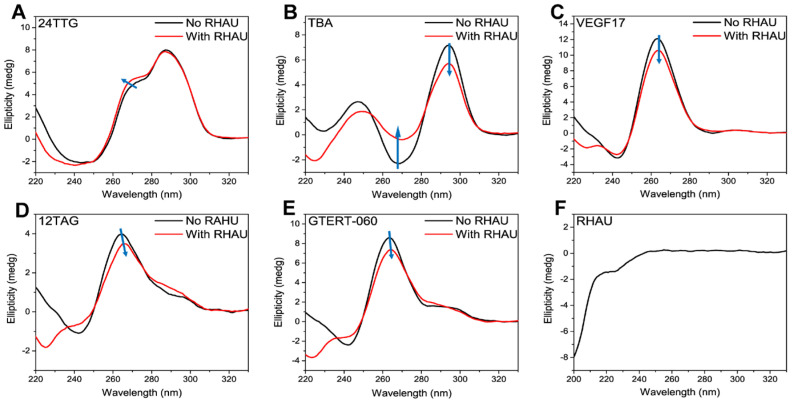
CD spectra of (**A**) 24TTG, (**B**) TBA, (**C**) VEGF17, (**D**) 12TAG and (**E**) GTERT-060 without (black lines) and with (red lines) RHAU at the ratio of 1:2 (10 μM G4s and 20 μM RHAU) after 30 min incubation at room temperature. (**F**) CD spectrum of RHAU (20 μM) dissolved in water. The blue arrows indicated the variation trend of CD signals from “No RHAU” to “With RHAU”.

Firstly, the formation of G4s was confirmed using CD measurement, a conventional method to estimate nucleic acids’ secondary structure. Different G4 topologies have respective characteristic CD signals. Parallel G4s show a characteristic positive peak at around 265 nm and a negative peak at around 240 nm, while antiparallel G4s have two positive peaks at around 240 nm and 295 nm, and a negative peak at around 265 nm [52,53]. For hybrid (3 + 1) G4s, they display two positive peaks at around 265 nm and 295 nm, and a negative peak at around 240 nm [54]. A special situation is that of a G4-forming sequence which folds into several different structures simultaneously, and the CD signals exhibit a mixed feature which often resembles that of hybrid G4s [4,5,6]. As shown, 24TTG (Figure 1A), 12TAG (Figure 1D) and GTERT-060 (Figure 1E) all exhibited characteristic signals corresponding to that of hybrid or mixed G4 structures. As reported, 24TTG folded into a hybrid (3 + 1) G4 [7], while 12TAG and GTERT-060 folded into mixed structures (parallel and antiparallel G4s for 12TAG [4], parallel and hybrid G4s for GTERT-060 [5]). On the other hand, the structures of TBA and VEGF17 were relatively single. TBA folded into antiparallel G4 (Figure 1B), and VEGF17 folded into parallel G4 (Figure 1C), both of which were in agreement with previous studies [55,56].

It is reported that some G4 ligands induce a conformational change when interacting with G4s [20,57,58], which will mislead the structural analysis. In order to confirm whether RHAU (N’-SMHPGHLKGREIGMWYAKKQGQKNKEAERQEAVVHMDEREE QIVQLLNSVQAK-C’, M_w_ = 6484.3) caused similar induction, we also performed CD measurements on RHAU (Figure 1F) and G4–RHAU mixtures (Figure 1A–E in red lines). As shown in Figure 1F, RHAU had no signals in the region of 240–330 nm, which was due to the negligible absorption of Trp and Tyr residues at the near-UV end [59]. Hence, the differences between G4s and G4–RHAU mixtures in the region of 240–330 nm were attributed to the changes in G4 structures induced by RHAU addition. No obvious change was observed for 24TTG upon RHAU addition (Figure 1A). For TBA, as shown in Figure 1B, although it still folded into antiparallel G4, the obviously changed CD signals reminded us that RHAU induced some changes in TBA G4, possibly in G-tetrad stacking or loop arrangement. The peak of VEGF17 at around 265 nm was slightly weakened upon RHAU addition without other signals appearing concomitantly (Figure 1C), implying the occurrence of a small change in G-tetrad stacking. 12TAG (Figure 1D) displayed a weakened signal at around 265 nm and a slightly enhanced signal at around 295 nm, which meant a small transition from parallel to antiparallel G4s induced by RHAU. In Figure 1E, the spectrum of GTERT-060 upon RHAU addition also demonstrated a weakened peak at around 265 nm, which quite resembled that of VEGF17, indicating a similar influence of RHAU on these two G4s. Although RHAU had different effects on distinct G4 structures, its addition induced no significant changes in CD spectra, indicating the main G4 structures were maintained.

**Figure 2:**
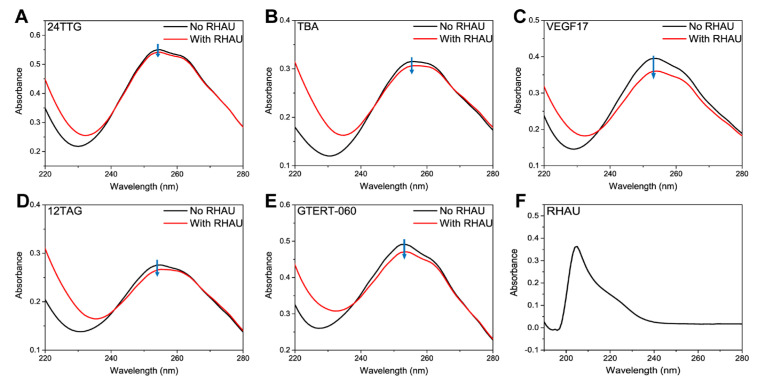
UV-Vis spectra of (**A**) 24TTG, (**B**) TBA, (**C**) VEGF17, (**D**) 12TAG and (**E**) GTERT-060 without RHAU (black lines) and with RHAU (red lines) at the ratio of 1:2 (10 μM G4s and 20 μM RHAU) after 30 min incubation at room temperature. (**F**) UV-Vis spectrum of RHAU (20 μM) dissolved in water. The blue arrows indicated the variation trend of UV-Vis signals from “No RHAU” to “With RHAU”.

DNAs have maximum UV-Vis absorptions at around 260 nm and changes in DNA structures lead to hypochromicity and red shift or blue shift of the spectra [60], which can also be used for studying changes in G4 structures. RHAU had no signals in the characteristic absorption range of G4s (Figure 2F), so the variation of absorption at around 260 nm should be entirely attributed to the perturbances of G4 structures. As shown, 24TTG (Figure 2A) had almost no UV changes upon RHAU addition, which indicated no structural change, consistent with CD results. Additionally, in Figure 2B–E, TBA, VEGF17, 12TAG and GTERT-060 displayed hypochromicity of different degrees, and VEGF17 had the biggest hypochromicity, meaning different reinforced stacking between G-tetrads induced by RHAU binding [61]. Overall, both CD and UV-Vis results showed that the effect of RHAU on various G4s was closely related to their structures, and the parallel G4 was influenced more obviously.

### 2.2. SEC Analysis

Based on the CD and UV-Vis measurements, the relative effect on G4 conformations can be observed after RHAU addition. However, it is still unable to accurately reflect the interaction between RHAU and the specific G4 structure. Therefore, SEC experiments were conducted on both G4s and G4–RHAU mixtures. In order to analyze the interaction between G4s and RHAU more conveniently, four different ratios of G4:RHAU of 1:0, 1:1, 1:2 and 1:4 were selected.

**Figure 3:**
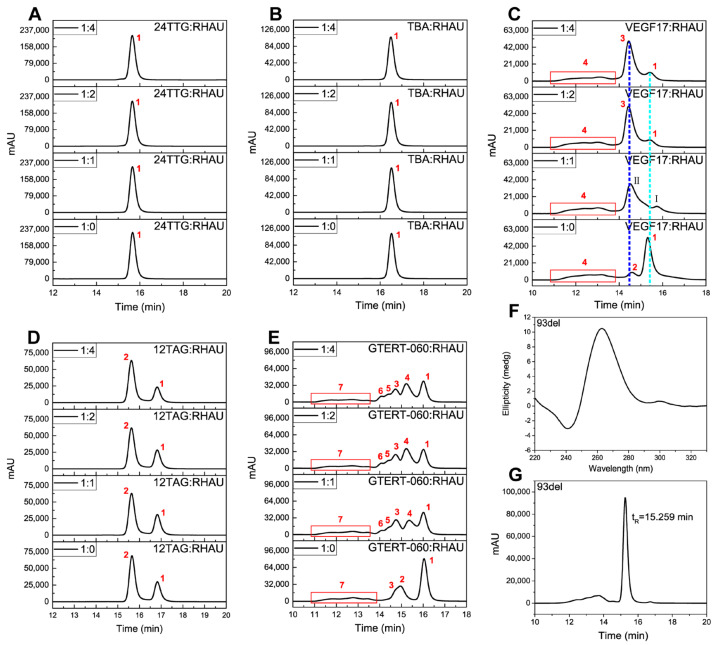
SEC chromatograms of G4s with RHAU in different molar ratios after 30 min incubation, and the CD spectrum and SEC chromatogram of 93del. (**A**) 24TTG, (**B**) TBA, (**C**) VEGF17, (**D**) 12TAG and (**E**) GTERT-060; ratios: 1:0, 1:1, 1:2 and 1:4. (**F**) CD spectrum and (**G**) SEC chromatogram of 93del dissolved in Tris-HCl buffer (5 mM Tris-HCl, 50 mM KCl, pH 7.4). Chromatograms were obtained under isocratic mode at the rate of 0.5 mL min^−1^ on a TSKgel G2000SW_XL_ column (7.8 × 300 mm i.d.) with 50 mM KH_2_PO_4_ as mobile phase. The concentrations of G4s were 10 μM. The column temperature was maintained at 30 °C and the detection wavelength was 260 nm. The chromatographic peaks of each G4 numbered in 1–7 corresponded to substances as described in Table 1. The light blue dashed line was used to distinguish the retention times between peak 1 and peak I, and the dark blue dashed line was used to distinguish the retention times between peak 2, peak 3 and peak II of VEGF17.

As presented in Figure 3A,B, 24TTG and TBA both had only one chromatographic peak at the ratio of 1:0, which were attributed to a hybrid G4 monomer [7] and antiparallel G4 monomer [56], respectively. The retentions of both of them stayed almost unchanged upon RHAU addition even when the ratio was adjusted to 1:4, corresponding to the condition that RHAU was greatly excessive. This phenomenon demonstrated that both hybrid 24TTG and antiparallel TBA had negligible interaction with RHAU. To better explain the chromatographic peaks for the rest of the G4s, a dimeric G4, 93del, was introduced. 93del had a predominant peak eluted at 15.259 min (Figure 3G), which was assigned to an interlocked bimolecular parallel G4 (Figure 3F [62]. The retention order of 24TTG, TBA and 93del was TBA > 24TTG > 93del, complying well with the retention rules of SEC. Therefore, the chromatograms of the remaining G4s were analyzed using 24TTG, TBA and 93del as relative standards based on the comparison of their molecular weights (M_w_) and retention times (*t*_R_).

For VEGF17 (Figure 3C) without RHAU (1:0), there was a predominant peak 1 with the longest *t*_R_, a small peak 2 with a relatively weaker retention and a bulge (peak 4) with a *t*_R_ between 11.1 and 14.0 min. CD spectra in Figure 1C and a previous study [55] both proved that VEGF17 folded into a parallel structure. Coupled with the comparison with 93del, peak 1 was assigned to a parallel G4 dimer. Upon RHAU addition (1:1), peak 1 was significantly reduced and two new peaks, peak I and peak II, appeared. However, when the ratio was adjusted to 1:2 and 1:4, there appeared a high and symmetrical peak 3 and a little of peak 1 remaining in chromatograms, indicating there was a transformation from peak 1 to peak 3. The transformation from peak 1 to peak 3 almost reached equilibrium at the ratio of 1:2, revealing that the parallel G4 was very easily reacted with RHAU. As to peak I and peak II, they were likely to be the unstable intermediates in the G4–RHAU interaction. It was noted that peak 2 disappeared upon RHAU addition, indicating that peak 2 may transformed to another peak or may be covered by peak 3. Based on the retention behaviors of VEGF17, as well as the comparison with 24TTG, TBA and 93del, the other peaks of VEGF17 and the VEGF17–RHAU mixture were deduced. Peak 3 was assigned to the complex of peak 1 and RHAU, namely the parallel dimer–RHAU complex. Meanwhile, it was noted that the retention time of peak 2 was weaker than peak 1 and slightly stronger than peak 3. So, it was reasonable to consider peak 2 as a parallel G4 trimer from the correlation between retention and molecular volume. Additionally, the bulge (peak 4) might be attributed to higher-ordered G4 structures, whose *t*_R_ was almost unchanged after adding RHAU.

For 12TAG (Figure 3D) without RHAU, there was a main peak 2. By being compared with 24TTG, TBA and 93del, peak 2 was assigned to the mixtures consisting of double-stranded parallel and antiparallel G4s [4]. Different from 24TTG, TBA and VEGF17, there was another prominent peak 1 for 12TAG. The longer *t*_R_ of peak 1 than peak 2 indicated a relatively lower molecular weight or more compact structure for peak 1. Upon the addition of RHAU, peak 1 and peak 2 stayed almost the same whatever the 12TAG:RHAU ratio was, meaning that the interaction between 12TAG and RHAU could be ignored, whereas it was observed that RHAU had some influence on the 12TAG structure as indicated by the CD results in Figure 1D. Additionally, according to what was observed for parallel VEGF17 (Figure 3C), the parallel component of 12TAG was also supposed to interact with RHAU. However, it was not reflected in SEC. This unexpected result was probably due to the intermolecular structure of 12TAG whose G4 monomers are double-stranded instead of single-stranded like VEGF17 G4. RHAU recognizes and binds to G4s via stacking on the terminal G-tetrad [39]. Therefore, more exposure of the terminal G-tetrad allows RHAU to better access G4s. The free bases around the terminal G-tetrads of intermolecular G4s resulted in greater steric hindrance than intramolecular G4s [63]. Consequently, we speculated that the large steric hindrance of terminal G-tetrads of intermolecular parallel G4 weakened the interaction between RHAU and 12TAG. Despite the 12TAG–RHAU interaction that was observed from CD, it was too weak to be maintained in SEC analysis, making it hard to detect the parallel G4–RHAU complex.

As for GTERT-060 (Figure 3E), another G4 of mixed structures, which folded into intramolecular G4s [5], things were a little different. Without RHAU, the predominant peak 1 was regarded as the monomeric mixture of parallel and hybrid G4s deduced in the same way as for VEGF17 and 12TAG. The much smaller peak at *t*_R_ = 14.5–15.5 min was asymmetrical, indicating that it contained more than one compound. Therefore, we assumed that it was the mixture of peak 2 and peak 3, which might have similar molecular weights. In addition, there was also a bulge (peak 7) at *t*_R_ = 11.1–13.8 min similar to that in VEGF17. Since RHAU was inclined to react with parallel G4s, the significant reduction in peak 1 after RHAU addition (1:1) pointed out that there was a decrease for the parallel G4 monomer, and the remaining peak 1 was mainly attributed to the hybrid G4 monomer. At the same time, a new peak 4 emerged, which was attributed to the parallel monomeric G4–RHAU complex. Adjusting the ratio to 1:2, peak 1 continued to decrease and peak 4 continued to increase, representing more transformation from monomer to G4–RHAU complexes. It was also observed that at the ratio of 1:1, peak 2 disappeared and peak 3 was weakened. Meanwhile, new weakly retained peaks, peak 5 and peak 6, appeared. Peak 3 showed a continuous decline at the ratio of 1:2, while peak 6 increased to some extent. Based on the difference in *t*_R_ and change trend, it was reasonable to speculate that peak 2 and peak 3 were both parallel G4 dimers of slightly different structural features, which were transformed to peak 5 and peak 6, respectively, upon RHAU addition. When adjusting the ratio to 1:4, the intensity of all the peaks was almost the same as that obtained at the ratio of 1:2, meaning that the binding of RHAU to GTERT-060 achieved saturation at 1:2. Compared with the intermolecular 12TAG G4, intramolecular GTERT-060 G4 has much less steric hindrance around terminal G-tetrads, leading to easier interaction with RHAU and finally to different retention behaviors. From the comparison of intermolecular parallel 12TAG and intramolecular parallel GTERT-060, it was concluded that steric hindrances of the terminal G-tetrads, i.e., the accessibility of RHAU to the terminals, rather than G4 topologies, played a predominant role in RHAU binding. Overall, from the chromatographic experiments, we knew that RHAU had specific binding ability to intramolecular parallel G4s, causing the weakened retention of corresponding peaks.

**Figure 4:**
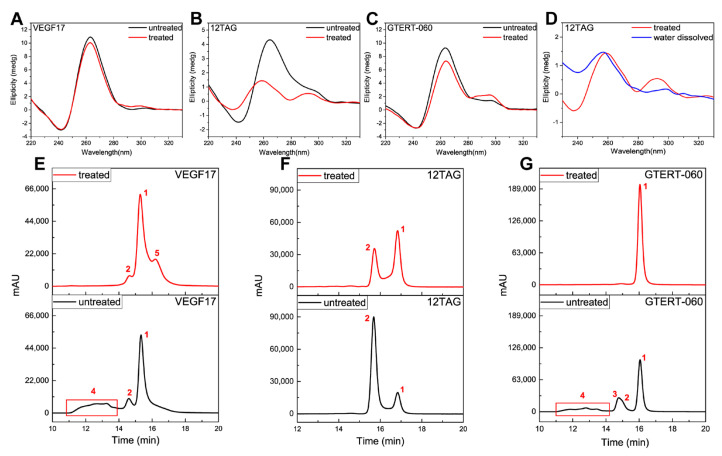
CD spectra and SEC chromatograms of (**A**,**E**) VEGF17, (**B**,**D**,**F**) 12TAG and (**C**,**G**) GTERT-060 without and with chemical treatments. Chromatograms were obtained under isocratic mode at the rate of 0.5 mL min^−1^ on a TSKgel G2000SW_XL_ column (7.8 × 300 mm i.d.) with 50 mM KH_2_PO_4_ as mobile phase. The concentrations of G4s were 10 μM. The column temperature was maintained at 30 °C and the detection wavelength was 260 nm. Chemical treatment condition: treated G4s with KOH solution (0.5 M) for 5 min at room temperature, and then added HCl solution (0.5 M) to return the pH to neutral. The black line in (**B**) represented annealed 12TAG G4 without chemical treatment, and the red lines in (**B**,**D**) represented annealed 12TAG G4 after chemical treatment. The blue line in (**D**) represented unannealed 12TAG which was directly dissolved in water and in ssDNA status.

Hereto, the assignment of some peaks in VEGF17, 12TAG and GTERT-060 was still uncertain, especially for those hypothetical multimers. Studies have proposed that chemical treatment can destroy G4 multimers and the content of multimers is expected to become minimum after treatment because of the disruption and the refolding of G-tetrads [51,64]. Hence, to better assign these uncertain peaks, G4s were chemically treated as follows: we first treated G4s with KOH solution (0.5 M) for 5 min at room temperature, and then added HCl solution (0.5 M) to return the pH to neutral. CD results in Figure 4A–C illustrate the effect of chemical treatment on G4 structures. It was seen that the main structure of VEGF17 was kept (Figure 4A). For 12TAG, after chemical treatment, the signal at 265 nm decreased dramatically and blue-shift took place, meaning an obvious change in structure (Figure 4B). Through further comparison with water-dissolved 12TAG (Figure 4D), which did not fold into a G4 structure, it was believed that the parallel G4 of 12TAG was dissociated into single-stranded DNA (ssDNA). As to GTERT-060 (Figure 4C), the signal changes after chemical treatment indicated there was a decrease in parallel structure and an increase in hybrid structure simultaneously.

After chemical treatment, VEGF17, 12TAG and GTERT-060 were analyzed under the same chromatographic conditions as that of untreated G4s. For VEGF17 (Figure 4E), peak 4 almost disappeared after treatment, verifying that it was the G4 multimers as we deduced. Peak 1 and peak 2 stayed almost unchanged, meaning that their corresponding substances were stable enough to resist a strong alkali environment. The emerging peak 5 was possibly a parallel monomeric G4 derived from multimer dissociation. After chemical treatment, peak 1 of 12TAG (Figure 4F) was obviously enhanced, while peak 2 was reduced, indicating a transformation between peak 1 and peak 2. Combining with the CD results in Figure 4B,D, peak 1 was believed to be ssDNA, and peak 2 was the mixture of double-stranded parallel and antiparallel G4s, consistent with the deduction for untreated 12TAG. After being treated, the parallel G4 of peak 2 was transformed to ssDNA, which was loosely structured with a longer retention. The remaining peak 2 was mainly attributed to the antiparallel G4 as indicated by the small peak at around 295 nm in Figure 4B (red line). Shown in Figure 4G, after chemical treatment, the main peak 1 of GTERT-060 was enhanced dramatically, while peak 2, peak 3 and peak 4 all disappeared. From Figure 4C, we knew that the conformation of GTERT-060 was kept all the time. Therefore, the disappeared peaks should all be parallel structures and almost all of them were transformed to monomers after chemical treatment, which was indicated by the significantly enhanced peak 1 corresponding to monomers. Based on *t*_R_, peak 2 and peak 3 were attributed to a G4 dimer with different structural features as deduced in Figure 3E, and the weakest retention of peaks 4 was assigned to higher-ordered G4s.

From above, peak 1 of VEGF17, and peak 2 and peak 3 of GTERT-060 were all ascribed to dimers. However, these three peaks behaved differently after being chemically treated. Peak 1 of VEGF17 stayed almost the same, while peak 2 and peak 3 of GTERT-060 disappeared, implying that these three dimers had different stability. There are several forms for G4 dimers. One is that two G4 monomers stack on each other via the π-π stacking of terminal G-tetrads (stacked dimer). The second is the formation of two tandem G4s within one nucleic acid strand (“beads-on-string” or tandem dimer). The third is that two G4 monomers interlock with each other where the guanine of one monomer pairs with that of the other monomer to complete the G-tetrad formation (interlocked dimer). Since the sequence compositions of VEGF17 and GTERT-060 were not capable of forming tandem dimers, their dimers were deduced to be stacked or interlocked ones. Since an interlocked dimer is more stable than a stacked dimer, the peak 1 of VEGF17 was therefore deduced to be and interlocked dimer, and peak 2 and peak 3 of GTERT-060 were deduced to be stacked dimers.

The assignments of chromatographic peaks for G4s before and after RHAU addition presented in Figure 3 were summarized in Table 1. From the SEC results, it was concluded that most G4–RHAU mixtures reached equilibrium state when the ratio of G4:RHAU was 1:2. Therefore, we chose 1:2 as the optimal ratio for other experiments.

**Table 1:** Chromatographic peaks assignments of studied G4s presented in Figure 3.

G4s	Molecular Weight (M_w_)	Peaks	*t*_R_ (min)	Corresponding Substances
24TTG	7563.97	Peak 1	15.668	hybrid monomer
TBA	4805.10	Peak 1	16.522	antiparallel monomer
VEGF17	5522.5	Peak 1	15.316	parallel dimer
		Peak 2	14.586	parallel trimer
		Peak 3	14.434	parallel dimer + RHAU complex
		Peak 4	11.1–14.0	parallel multimers
12TAG	3835.49	Peak 1	16.822	ssDNA
		Peak 2	15.655	bimolecular monomers mixture (parallel and antiparallel)
GTERT-060	6448.14	Peak 1	16.045	monomers mixture (parallel and hybrid)
		Peak 2	14.949	parallel dimer
		Peak 3	14.721	parallel dimer
		Peak 4	15.356	parallel monomer + RHAU complex
		Peak 5	14.422	parallel dimer + RHAU complex
		Peak 6	14.107	parallel dimer + RHAU complex
		Peak 7	11.1–13.8	parallel multimers

### 2.3. Native-PAGE Analysis

**Figure 5:**
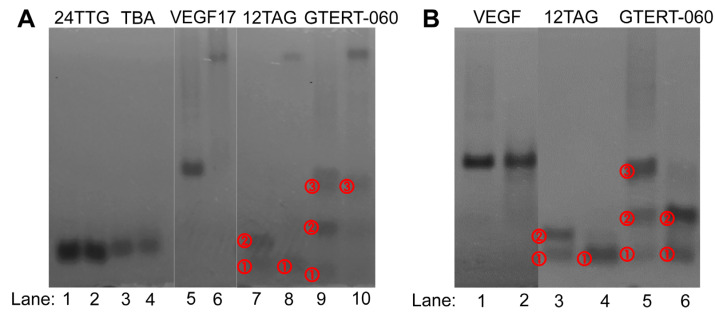
(**A**) Native-PAGE electrophoretograms of 24TTG (lanes 1, 2), TBA (lanes 3, 4), VEGF17 (lanes 5, 6), 12TAG (lanes 7, 8) and GTERT-060 (lanes 9, 10) without and with RHAU addition. Lanes 1, 3, 5, 7 and 9 represented individual G4s and lanes 2, 4, 6, 8 and 10 represented G4–RHAU mixture at a molar ratio of 1:2 after 30 min incubation at room temperature. (**B**) Native-PAGE electrophoretograms of VEGF17 (lanes 1, 2), 12TAG (lanes 3, 4) and GTERT-060 (lanes 5, 6) before and after chemical treatment. Lanes 1, 3 and 5 represented G4s before chemical treatment and lanes 2, 4 and 6 represented G4s after chemical treatment. The migrating bands of each G4 were numbered in ①−③ to be more identifiable. Note: the images were reconstituted and the lanes were rearranged to keep the order of G4s consistent with those in Figure 1, Figure 2, Figure 3 and Figure 4.

Native-PAGE experiments were performed to help verify the SEC results. As shown in Figure 5A, without RHAU, hybrid 24TTG and antiparallel TBA displayed only one migrating band (lanes 1 and 3), corresponding to the monomer G4s (peaks 1 in Figure 3A,B). Upon RHAU addition, the bands of 24TTG and TBA were unchanged (lanes 2 and 4), demonstrating no interaction with RHAU, which was well consistent with results in the SEC experiments. Parallel VEGF17 (lane 5) showed a predominant migrating band, which was attributed to the dimer as deduced for peak 1 in Figure 3C. After the addition of RHAU, a new band with a very slow migration rate appeared (lane 6), corresponding to a G4–RHAU complex (peak 3 in Figure 3C), which indicated the occurrence of the interaction between VEGF17 G4 and RHAU. There were almost no other obvious migrating bands in lane 6 except the one corresponding to a G4–RHAU complex, suggesting a nearly complete transformation from VEGF17 to a VEGF17–RHAU complex.

For 12TAG, similar to the SEC result, there were two main bands which migrated close to each other in lane 7 in Figure 5A. The fast migrating band ① corresponded to the ssDNA (peak 1 in Figure 3D) whose migrating rate did not change upon RHAU addition (lane 8, band ①). The slow migrating band ② was thought to be the mixture of parallel monomers and antiparallel monomers (peak 2 in Figure 3D). It was worth noting that there was a much slower and light-colored migrating band in lane 8, corresponding to a G4–RHAU complex. That was to say, for 12TAG, the interaction with RHAU was observed in Native-PAGE, which was different from that in SEC. Although the band of a G4–RHAU complex was observed, since 12TAG G4 was composed of parallel and antiparallel G4s, there should still exist a light-colored band corresponding to the remaining antiparallel G4 in lane 8, for the reason that antiparallel G4 had no interaction with RHAU. However, there did not. It might be because the content of the remaining antiparallel G4 was not enough to be detected. Different from what was observed in SEC, GTERT-060 had three main bands in lane 9, of which band ② represented the mixture of parallel and hybrid monomers (peak 1 in Figure 3E), and band ③ corresponded to the mixture of two dimers (peak 2 and peak 3 in Figure 3E). Additionally, in the forefront of lane 9, there was an additional light-colored band ①. This band might be ssDNA which was co-eluted with monomers in peak 1 in SEC (Figure 3E). Upon RHAU addition, the obvious slow-migrating band in lane 10 confirmed the formation of a G4–RHAU complex, corresponding to the parallel monomer–RHAU complex (peak 4 in Figure 3E). In addition, in lane 10, the remaining band ③ corresponded to peak 3 in Figure 3E, and the disappearance of band ① implied a transformation from ssDNA to G4 and then to a G4–RHAU complex.

VEGF17, 12TAG and GTERT-060 after chemical treatment were also analyzed using Native-PAGE (Figure 5B). As shown in lanes 1 and 2, the main migrating band of VEGF17 stayed the same, which was consistent with the peak 1 in Figure 4E. For 12TAG, after the treatment, only band ① remained in lane 4, which verified the decrease in G4 monomer and the increase in ssDNA as described in SEC. Similarly, for GTERT-060, the same migrating but deeper-colored band ① and band ② in lane 6 well corresponded to peak 1 in Figure 4G which had an unchanged *t*_R_ but a small enhancement in intensity.

Although there were some subtle differences, the results of Native-PAGE and SEC basically corroborated each other. Native-PAGE seemed to display better separation upon RHAU addition than SEC based on the fact that the bands of G4–RHAU complexes were far away from those of free G4s. However, for Native-PAGE analysis, high G4 concentration is needed, which can cause misleading results based on the fact that the G4 structures formed in high DNA concentrations might be different from those formed at low DNA concentrations, giving HPLC unique practical significance. In addition, it was found that Native-PAGE lost a lot of detail, such as the absence of multimers of VEGF17 and GTERT-060 observed in SEC. Furthermore, the operation for HPLC was simpler and rapid, and the reproducibility was better than Native-PAGE. In this way, HPLC can provide some unique perspectives to more easily and better understand the interaction between ligands and various G4 structures.

## 3. Materials and Methods

### 3.1. Materials and Reagents

The oligonucleotides (listed in Table 2) and the 55-amine-acid RHAU peptide (N’-SMHPGHLKGREIGMWYAKKQGQKNKEAERQERAVVHMDEREEQIVQLLNSVQAK-C’) were all custom-synthesized by Sangon Biotech Co., Ltd. (Shanghai, China). The water used was ultrapure water. All of other chemicals were of analytical reagent grade unless otherwise noted.

### 3.2. Sample Preparation

Single-stranded oligonucleotides were dissolved in Tris-HCl buffer (10 mM Tris-HCl, 100 mM KCl, pH 7.4) and quantified using UV-Vis absorption spectroscopy with the following extinction coefficients (ε_260_ nm, M^−1^ cm^−1^) for each nucleotide: A = 15,400, G = 11,500, C = 7400 and T = 8700. The DNA solutions were heated in a 95 °C water bath for 5 min and cooled to room temperature naturally to prepare the G4s. The cooled solutions were then kept at 4 °C overnight to be stock solutions followed by being stored at −80 °C before use. RHAU was dissolved in ultrapure water and also stored at −80 °C. Before use, the stock solution of RHAU was diluted to the required concentration with ultrapure water. G4s to be measured were prepared by mixing G4 solution of a desired concentration with an equal volume of water. For G4–RHAU mixtures, the stock solutions of RHAU and G4s were diluted to the needed concentrations, and then were mixed in equal volumes followed by being incubated for 30 min before being measured.

### 3.3. CD Experiments and UV-Vis Absorption Spectroscopy

CD measurements were performed using a Chirascan digital circular dichroism spectropolarimeter (Applied Photophysics Ltd., Leatherhead, UK) and a 1 mm path length quartz cuvette. The sampling interval was 0.5 s and the slit width was 1 nm. The resulting measurements were the average of three repetitions between 220 nm and 330 nm at room temperature. The CD spectra of the baseline and the buffer were subtracted from the spectra of the G4 solutions. All G4s for CD measurements were prepared at the concentration of 10 μM. For the G4–RHAU mixtures, the concentrations of G4 and RHAU were set at 10 μM and 20 μM, respectively.

UV-Vis absorptions were performed using a UH5300 UV-Vis spectrophotometer (Hitachi, Tokyo, Japan) and a 1 cm path length quartz cuvette. Absorbance was measured from 190 nm to 350 nm at room temperature. The UV-Vis spectrum of the background was subtracted from the spectrum of each G4 solution. G4s for UV-Vis measurements were prepared at a concentration of 2 μM. For the G4–RHAU mixtures, the concentrations of G4 and RHAU were set at 2 μM and 4 μM, respectively.

### 3.4. SEC Conditions

SEC experiments were performed on an LC-20AD (Shimadzu, Kyoto, Japan) with a UV-Vis detector. Separation was accomplished on a TSKgel G2000SW_XL_ column (7.8 × 300 mm i.d.). The column temperature was maintained at 30 °C, and the detection wavelength was set at 214 nm for RHAU and 260 nm for G4s and G4–RHAU mixtures. KH_2_PO_4_ (50 mM, pH 7.0) buffer solution was used as mobile phase for isocratic elution at a flow rate of 0.5 mL min^−1^. The final concentration was 10 μM for different G4s with an injection volume of 10 μL, and the ratio of G4:RHAU was set at 1:0, 1:1, 1:2 and 1:4.

### 3.5. Native-PAGE Experiments

Native-PAGE experiments were carried out on a Biorad PowerPacTM HV apparatus (Bio-Rad, Hercules, CA, USA). G4s and RHAU were prepared at the concentrations of 50 μM and 100 μM, respectively, to provide clear bands for analysis. Electrophoresis was performed using a 20% polyacrylamide gel, containing 50 mM KCl and 1 × TBE buffer (80 mM Tris-borate, 2 mM EDTA, pH 8.3). G4s and G4–RHAU mixtures ran in 1 × TBE buffer, supplemented with 50 mM KCl using the following parameters: ice-water bath, voltage = 120 V and time = 2 h. Bands in the gels were visualized by UV-shadowing.

## 4. Conclusions

With the increasing findings of G-quadruplexes in disease-related genes, there is growing interest in the exploitation of ligands that can specifically target G4s in order to seek new therapeutic agents for diseases. Therefore, the study of the interaction between ligands and G4s has become increasingly significant, which promotes the development and discovery of new specific ligands with therapeutic effect. RHAU plays an important role in many important biological processes by mediating G4s, making it a biologically important G4 ligand. However, the knowledge about the interaction between RHAU and G4s with different conformations is relatively lacking. In this article, we studied the SEC chromatographic retention behaviors of five G4-forming sequences with different secondary structures, and their interactions with RHAU peptide were also elucidated using SEC. RHAU peptide selectively weakened the chromatographic retention of parallel G4s without changing the main structures, demonstrating a specific targeting ability. The conformational selectivity exhibited by RHAU peptide in SEC experiments was verified by Native-PAGE, which further confirmed the reliability of SEC for studying the G4–peptide interactions. Interestingly, we also found that the RHAU peptide interacted differently with intermolecular parallel and intramolecular parallel G4s, which suggested that steric hindrance was the main factor affecting RHAU-binding ability rather than the G4 conformation. Conclusively, this study enriched information on the interactions of RHAU and G4s with different secondary structures, which will contribute to facilitate the deeper understanding of how RHAU exerts its biological functions. Importantly, the inherent separation ability of SEC to investigate G4–peptide interactions, especially for a multiple G4 coexisting system, endows its applicability in screening for novel G4 ligands.

## Figures and Tables

**Table 2:** DNA sequences utilized in this research.

Names	DNA Sequences (5′ to 3′)	Description	G4 Conformations
24TTG	TTG GGT TAG GGT TAG GGT TAG GGA	Human telomere	Hybrid monomer [7]
TBA	GGT TGG TGT GGT TGG	Thrombin-binding aptamer	Antiparallel monomer [56]
VEGF17	GGG AGG GTT GGG GTG GG	Human vascular endothelial growth factor proximal promoter	Parallel [55]
12TAG	TAG GGT TAG GGT	Human telomere	Double-stranded parallel and antiparallel [4]
GTERT-060	AGG GGA GGG GCT GGG AGG GC	Human telomerase, hTERT promoter	Parallel and hybrid [5]
93del	GGG GTG GGA GGA GGG T	Aptamer, inhibitor of HIV-1 integrase	Interlocked bimolecular parallel [62]

## Data Availability

Not applicable.
